# Multi-Parameter Laser Imaging Reveals Complex Microscale Biofilm Matrix in a Thick (4,000 μm) Aerobic Methanol Oxidizing Community

**DOI:** 10.3389/fmicb.2018.02186

**Published:** 2018-09-19

**Authors:** John R. Lawrence, Marcus Winkler, Thomas R. Neu

**Affiliations:** ^1^Environment and Climate Change Canada, Saskatoon, SK, Canada; ^2^Waters Corporation, Eschborn, Germany; ^3^Department of River Ecology, Helmholtz Centre for Environmental Research, Magdeburg, Germany

**Keywords:** biofilms, microenvironment, methanol, EPS, FISH, CLSM

## Abstract

Although methanol has frequently been used as an inexpensive supplementary carbon source to support treatment processes, knowledge of the resultant microbial biofilms, their 3D architecture, microenvironments, exopolymer chemistry and populations remains limited. We supplied methanol as a supplementary carbon source to biofilms developing in rotating annular reactors. Analysis of circulation waters (1.0 l d^−1^) indicated that dissolved organic carbon was reduced by 25%, NO_3_-nitrogen by 95%, and total phosphorus by 70%. Analyses of populations using culture based techniques and fluorescence *in situ* hybridization indicated enrichment of nitrifiers, denitrifiers, and methylotrophic bacteria relative to reference biofilms not receiving methanol. The biofilms that developed were up to 4,000 μm thick. Staining with fluor conjugated lectins in combination with nucleic acid stains, revealed the presence of discrete bacterial cells inside complex globular polymeric structures. These structures were in turn surrounded by an interstitial polymer containing a variety of bacterial cell types. The globular structures bound FITC-conjugated lectins, from *Canavalia ensiformis* and *Ulex europeaus*. The FITC-lectin of *Phaseolus vulgaris* bound the surface of the globular structures and more generally within the matrix. Chemical analyses of the polymer paralleled the results of lectin analyses indicating that the dominant neutral sugars were glucose, galactose, mannose, rhamnose, with fucose and ribose as minor constituents. Amino sugars were not detected. Dual channel imaging with pH sensitive probes indicated that pH gradients from pH 4 to 7 occurred across the globular microcolonies. Critically for the maintenance of aerobic conditions throughout the thick biofilm it was extensively penetrated by a fine fissure network revealed by the location of fluorescent latex microbeads as detected by confocal laser scanning microscopy. Microelectrode studies confirmed the absence of any detectable Eh gradients within the biofilm. However, mobility of various size-fractionated fluorescent probes indicated that the basal region was only penetrated by the lowest molecular weight probes with a hydrated radius of 2.2 nm or less. These observations indicate the selection of a unique, thick (>4,000 μm) microbial community in which a self-organized architecture promotes the maintenance of optimal conditions and metabolism throughout the biofilm community.

## Introduction

Methanol is generated in a variety of industrial processes, including, pulp mills, and coal gasification (Mohr and King, 1985; Minami et al., 1986). In addition, it is commonly found in a wide range of natural anaerobic habitats (Schink and Zeikus, 1982). It is a simple C1 compound that may support a wide range of physiological groups and an extensive number of trophic levels. Methanogenic and acetogenic bacteria are able to utilize methanol as a carbon and energy source, nitrate and sulfate reducing bacteria may also utilize methanol (Florencio et al., 1994). Given these attributes of availability, utility as carbon and energy source, and capacity to support a range of physiological processes, methanol makes a useful supplemental carbon source. In industrial settings methanol has frequently been used as an inexpensive supplementary carbon source to support waste water treatment processes such as post denitrification. It has also been used to assist the combined removal of phosphorus and nitrogen from waste streams (Nyberg et al., 1992). These industrial scale reactors frequently use sand as a matrix to support development of an extensive biofilm community (Neef et al., 1996). Microsensors and imaging have been used to demonstrate steep oxygen and pH gradients in a variety of aerobic biofilm systems indicating the importance of diffusion processes and structure in biofilm systems (Dalsgaard and Revsbech, 1992; DeBeer et al., 1994; Lawrence et al., 2016). A variety of confocal microscopy studies have also confirmed the importance of cell distribution patterns, void spaces and channels in the architecture of degradative biofilm communities (Wolfaardt et al., 1994; Massol-Deyá et al., 1995; Macedo et al., 2005, 2007; Wouters et al., 2010). Neef et al. (1996) carried out an analysis of a methanol fed biofilm concentrating on the application of *in situ* hybridization to define populations of bacteria present in the biofilm community. However, knowledge of the development of methanol fed microbial biofilms, their architecture, exopolymer chemistry, and micro environmental conditions, is limited. Mass spectrometry was used to assess the nature and abundance of neutral sugars in the polymer matrix. Further, conventional culture media were used to determine plate counts and most probable numbers of a range of physiological types of microorganisms. We applied, confocal laser scanning microscopy (CLSM) in conjunction with a range of fluor-conjugated molecular probes for non-destructive analyses of the biofilms. In particular, size fractionated probes, charged probes, microelectrodes, glycoconjugate specific lectins and other specific fluorescent probes to address whether architectural arrangements in a thick (>4,000 μm) biofilm community would influence environmental gradients.

## Materials and methods

### Reactors

Biofilms were cultivated in a simple rotating annular reactor (Lawrence et al., 2000) with river water as a source of inoculum and supplemented with 200 mg l^−1^ of methanol as a source of carbon. Reference reactors without methanol addition were also run in parallel. The water was pumped through the reactors at a rate of 500 ml per day (one reactor volume) by using a multichannel peristaltic pump (Watson Marlow, Wilmington, Mass.). Reactor cores with 12 replicate removable strips for examination of biofilms were constructed of polycarbonate. The reactors were run in a recirculation modethe main reservoir was refilled with fresh river water and 200 mg l^−1^ of methanol added every 7 days.

### Confocal laser microscopy and probes

The biofilm was characterized using a number of analytical imaging techniques. Confocal laser scanning microscopy was performed with either an MRC 1024 CLSM (Biorad, Hemel Hempstead, UK) attached to a Microphot SA microscope (Nikon, Tokyo, Japan) and the following water immersible lenses 63x, 0.9 NA (Zeiss, Jena, Germany) and 40x, 0.55 NA (Nikon), or the TCS-SP1 (Leica, Heidelberg, Germany) controlled by the Confocal Software Version 2.00 Build 0477 and attached to an upright microscope (Leica), equipped with 20x, 0.5 NA, 40x, 0.8 NA, 63x, 0.9 NA, and 63x (water immersion) 1.2 NA lenses. In addition, observations were made using the 2-Photon mode of the TCS-SP1 system, equipped with a pulsed pico second infrared laser. Details of 2-Photon-LSM are discussed elsewhere (Neu et al., 2002; Neu and Lawrence, 2015). Routine analyses of the biofilms for bacteria, photosynthetic biomass and general glycoconjugates were carried out using the triple imaging procedure of Lawrence et al. (2000). Bacterial biomass was determined using Syto9 staining in the green channel, photosynthetic biomass was determined by autofluorescence in the far red and the *Triticum vulgaris* lectin was used with a TRITC label to detect and quantify lectin-specific matrix glycoconjugates (Neu et al., 2001, 2004). These analyses were carried out routinely through 160 days of development after which the methanol system reached a steady state and overall biofilm thickness precluded this type of routine analyses.

Detailed analyses of biofilm structures were carried out using 1-photon and 2-photon laser scanning microscopy following staining with a range of probes (Table 1) nucleic acid stains Syto 9, the protein stain SyproOrange, (Thermo Fisher Scientific, Mississauga, Ontario, Canada) and a panel of fluor conjugated lectins. The panel included the following fluor conjugates: *Arachis hypogaea* CY5, *Canavalia ensiformis* (tetramethyl rhodamine isothiocyanate (TRITC), *Tetragonolobus purpureas* (CY5), *Helix aspersa-* fluorescein isothiocyanate (FITC) and *Caragana arborescens-FITC, Arachis hypogaea* FITC, *Canavalia ensiformis* FITC, *Glycine max* FITC, *Lens culinaris* FITC, *Limulus polyphemus* FITC, *Phaseolus vulgaris* FITC, *Tetragonolobus purpureas* FITC, *Triticum vulgaris* FITC, *Ulex europaeus* FITC, *Vicia faba* FITC, *Vicia villosa* FITC, *Wisteria floribunda* FITC were used to assess the distribution of glycoconjugates at the microscale (Neu et al., 2001). The fluorescent probe Nile Red was used to examine the distribution of hydrophobic regions in the biofilms. In addition, fluor conjugated 10K g mol^−1^ / molecular weight, anionic, polyanionic and neutrally charged dextrans were used to detect the distribution of charge in the matrix (Wolfaardt et al., 1998; Lawrence et al., 2016). Fluorescent 0.2 μm beads (Thermo Fisher Scientific, Mississauga, Ontario, Canada) and size fractionated dextrans (2000K, 400K. 40K, and 4K) conjugated to fluorescein isothiocyanate or rhodamine B were used to assess permeability of the biofilm matrix (Lawrence et al., 1994, 2016). Probes were prepared as described in Lawrence et al. (1994). The solutions were applied to the biofilm surface, incubated for 1–24 h and imaged using CLSM to assess the distribution of the probes within the biofilm. In the case of charge distribution analyses, the biofilms were rinsed 3X with filter-sterilized (0.2 μm) river water prior to imaging. Molecular weight (mw or g mol^−1^) and radius of gyration (nanometers) of size-fractionated probes are as follows, fluorescein, 289 mw, 0.98 nm; dextran, 4,000 mw, 2.2 nm; dextran, 40,000 mw, 4.65 nm; dextran, 70,000 mw, 9.02 nm; dextran, 400,000 mw, 10.5 nm; dextran, 2,000,000 mw, 57.9 nm respectively. Fluorescein was also applied as a negative stain as described by Caldwell et al. (1992).

**Table 1:** Binding of selected fluor conjugated lectins and other probes to the biofilm.

**Lectins and other probes**	**Globules**	**Matrix**
*Arachis hypogaea* CY5	–	–
*Arachis hypogaea* FITC	+	+
*Arachis hypogaea* TRITC	–	–
*Canavalia ensiformis* FITC	+	+
*Canavalia ensiformis* TRITC	–	–
*Caragana arborescens* TRITC	–	–
*Erythrinia cristigalli* TRITC	+	+/–
*Glycine max* FITC	–	–
*Glycine max* TRITC	–	–
*Lens culinaris* FITC	–	+
*Lens culinaris* TRITC	–	–
*Limulus polyphemus* FITC	–	–
*Phaseolus vulgaris* FITC	+	+
*Phaseolus vulgaris* TRITC	+	–
*Tetragonolobus purpureas* CY5	+	–
*Tetragonolobus purpureas* FITC	+	–
*Tetragonolobus purpureas* TRITC	–	–
*Triticum vulgaris* FITC	–	+
*Triticum vulgaris* TRITC	–	–
*Ulex europaeus* CY5	+	+
*Ulex europaeus* FITC	+	+
*Ulex europaeus* TRITC	+	–
*Vicia faba* FITC	–	+/–
*Vicia villosa* FITC	–	–
*Wisteria floribunda* FITC	–	+/–
SyproOrange	–	–
SyproRed	–	–
Dextran 10k m.w. anionic FITC	–	+
Dextran 10k m.w. polyanionic Rh B	–	+
Dextran 10k m.w. neutral Rh B	–	–
Nile Red	–	–

### Chemical analyses

The freeze dried exopolymer was also hydrolyzed and derivatized for subsequent chemical analysis of neutral carbohydrates and amino sugars using GC and GC-MS. Water chemistry was analyzed using standard methods as described by Environment Canada (1992).

### Microenvironmental conditions

Redox potential (Eh) measurements were made using platinum wire micro electrodes constructed as described by Swerhone et al. (1999). In brief, 99.99% pure Pt wire was cemented into glass micropipettes using epoxy, the tips were beveled using a micropipette beveller with #1500 abrasive paper and a copper wire connected to the Pt wire. Electrodes were mounted in a micromanipulator, and connected to a high impedance picoammeter to obtain readings in millivolts (mV). The electrodes were slowly inserted into the biofilm and readings taken at 10 μm intervals. Equilibration was allowed to occur for up to 20 min. Redox values were expressed as mV relative to a standard hydrogen electrode (American Public Health Association, 1992). A standard commercial Ag/AgC1, liquid, single junction reference electrode (Fisher Scientific) was used in all applications of the Pt wire electrodes. All electrodes were calibrated before and after use relative to a commercial redox electrode (Orion Research Inc., Cambridge, MA).

Detection of pH gradients within the methanol biofilms was performed using dual labeled rhodamine+fluorescein, 10K mw dextrans and ratiometric imaging procedures (Lawrence et al., 2016). A standard curve was created by equilibrating fixed (4% formaldehyde) biofilm samples with pH standards ranging from pH 4 to 9 and containing the Rh+Fl dextran. Dual channel imaging allowed simultaneous collection of images in the green and red wavelengths thus obtaining a pH insensitive fluorescent image (rhodamine) and a pH sensitive image (fluorescein). These images were then ratioed to correct for concentration variation and a standard curve relating gray value to pH created. Living biofilms were then imaged using the same rhodamine, fluorescein, labeled 10K mw dextran, and analyzed to assess the pH distribution within the methanol biofilms.

### Microbial populations

Biofilm subsamples (1 cm^2^) were prepared by scraping with sterile silicon spatulas to remove the biofilm and sonicated in a Bransonic 5120 water bath sonifier (Branson Ultrasonics, Danbury, Conn.) for 5 min to disperse the cells prior to dilution and subsequent plating and inoculation of a variety of media for both most probable number (MPN) and plate count estimation of specific populations. 10-fold dilutions were prepared in glass tubes containing 9 ml of sterile pH 7.0 river water. Dilutions were spread plated in triplicate onto 10% tryptic soy agar, actinomycete isolation agar, Rose Bengal agar and PTYG agar and then incubated at 23 ± 3°C under atmospheric oxygen. All dried media components were Difco Products (Thermo Fisher Scientific, Mississauga, Ontario, Canada). Counts of a variety of aerobic physiological groups were carried out using the MPN method and appropriate media. These included, thiosulfate oxidizers (Lawrence et al., 1991), nitrifying bacteria (Schmidt and Belser, 1982). Media were also inoculated to enumerate Fe III reducing populations (Lovley et al., 1990), sulfate reducing bacteria (Widdel and Bak, 1992), denitrifying bacteria (Tiedje, 1982) and methylotrophic bacteria (Raj, 1989).

### Fluorescent *in situ* hybridization

Fixation and hybridization were done following the protocol of Manz et al. (1999). Probes for *Paracoccus* were applied according to Neef et al. (1996). Oligonucleotide probes (EUB338, ALFlb, BET42a, GAM42A, CF319a, SRB 385Db, and *Paracoccus* (PAR651, PAR1244, PAR1457) (Interactiva, Berlin, Germany) were stored in TE buffer (10 mM Tris, 1 mM EDTA, pH 7.5) at−20°C. Working solutions were adjusted to 50 ng DNA per ml. Preheated hybridization buffer (0.9 M NaCl, 20 mM Tris/HCl [pH 7.2], 0.01 % SDS, formamide 35%) was mixed with fluorescently labeled oligonucleotide (1 ng ml^−1^ hybridization buffer) and applied to the fixed biofilm material. The slides were placed in 100% humidity chambers and incubated for 90 min at 46°C. After this, hybridization buffer was drawn off with tissue placed at the edges of the slides. Subsequently, slides were transferred to 50 ml preheated (48°C) washing buffer (20 mM Tris/HCl, 0.01% SDS, NaCl) and incubated at 48°C for 20 min. For microscopic analysis, slides were carefully rinsed with distilled water, air dried and mounted in antifading glycerol medium (Citifluor AF2, Citifluor Ltd., London, UK). All hybridization and washing steps were performed in the dark. Fixed biofilm samples were counter stained with SytoxGreen (Thermo Fisher Scientific, Mississauga, Ontario, Canada) for total cell counts. For digital analyses, images were individually thresholded and the percent area of hybridized or Sytox stained cells determined using the program NIH Image vl.64. Images with nonspecific binding or cross channel interference from algae or cyanobacteria were eliminated from the analyses. For group specific probes (ALFlb, BET42a, GAM42A, CF319a, SRB 385Db, and *Paracoccus* (PAR651, PAR1244, PAR1457) *n* = 14–20, whereas for EUB338 probe and total counts based on SytoxGreen staining *n* = 60.

### Experimental design

River biofilms were allowed to develop in the presence or absence of methanol. The reference and treatment reactors contained 12 identical coupons and each analysis was done on subsamples from randomly-selected biofilm coupons. CLSM imaging was done at a minimum of 5 random locations across a transect on the 1 cm^2^ piece of biofilm coupon. Subsampling for other analyses was also carried out using randomly-selected subsamples from among the 12 identical coupons in each reactor. Analysis of variance was used to detect significant differences among sample means at p < 0.05. Analyses were carried out using the commercial package, MiniTab (State College, PA, U.S.A).

## Results

### Biofilm development and architecture

**Figure 1:**
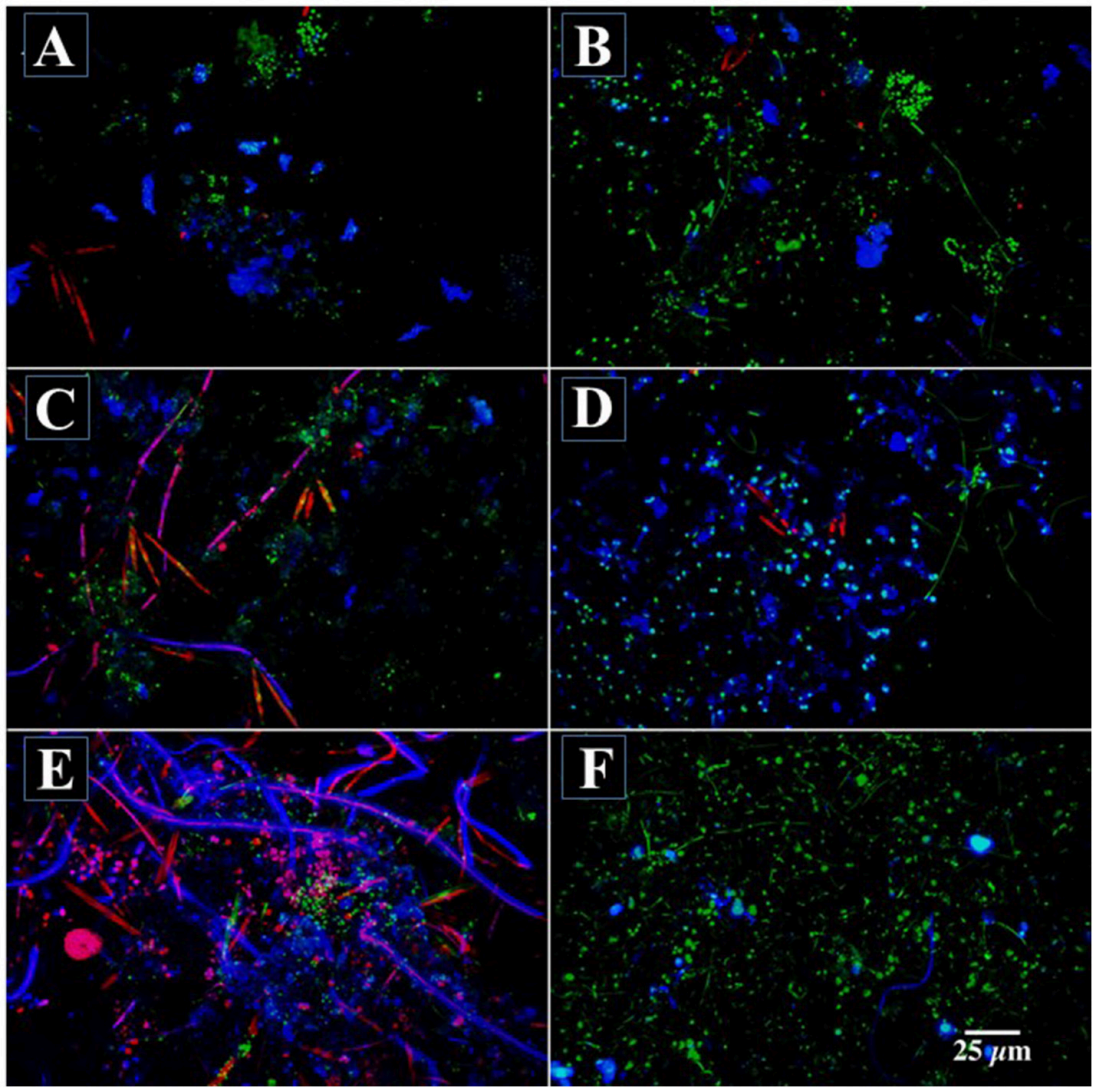
**(A–F)** Confocal laser micrographs showing the development of reference river biofilm **(A,C,E)** and river biofilm amended with methanol **(B,D,F)**, bacteria appear green, photosynthetic algae red and exopolymer blue. **(A–D)** shows the biofilm 4 days after initiation of methanol supplementation, **(B–E)** after 56 days and **(C–D)**, 87 days. The biofilm thickness at each time period was A = 60 μm, B = 80 μm, C = 170 μm, D = 100 μm, E = 170 μm, E = 430 μm respectively. Scale bar of 25 μm applies to all images.

**Figure 2:**
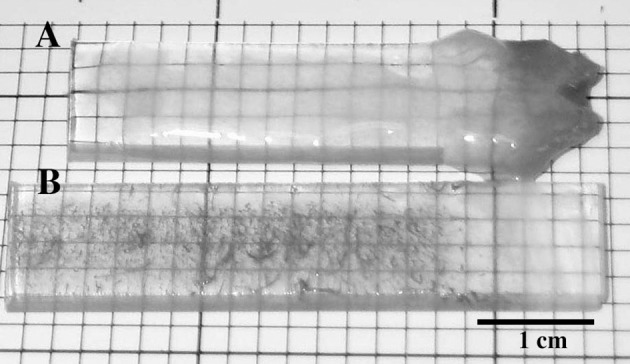
Photograph of the macroscopic appearance of the biofilm when grown on polycarbonate coupons **(A)** with and **(B)** without 200 mg l^−1^ methanol addition.

**Figure 3:**
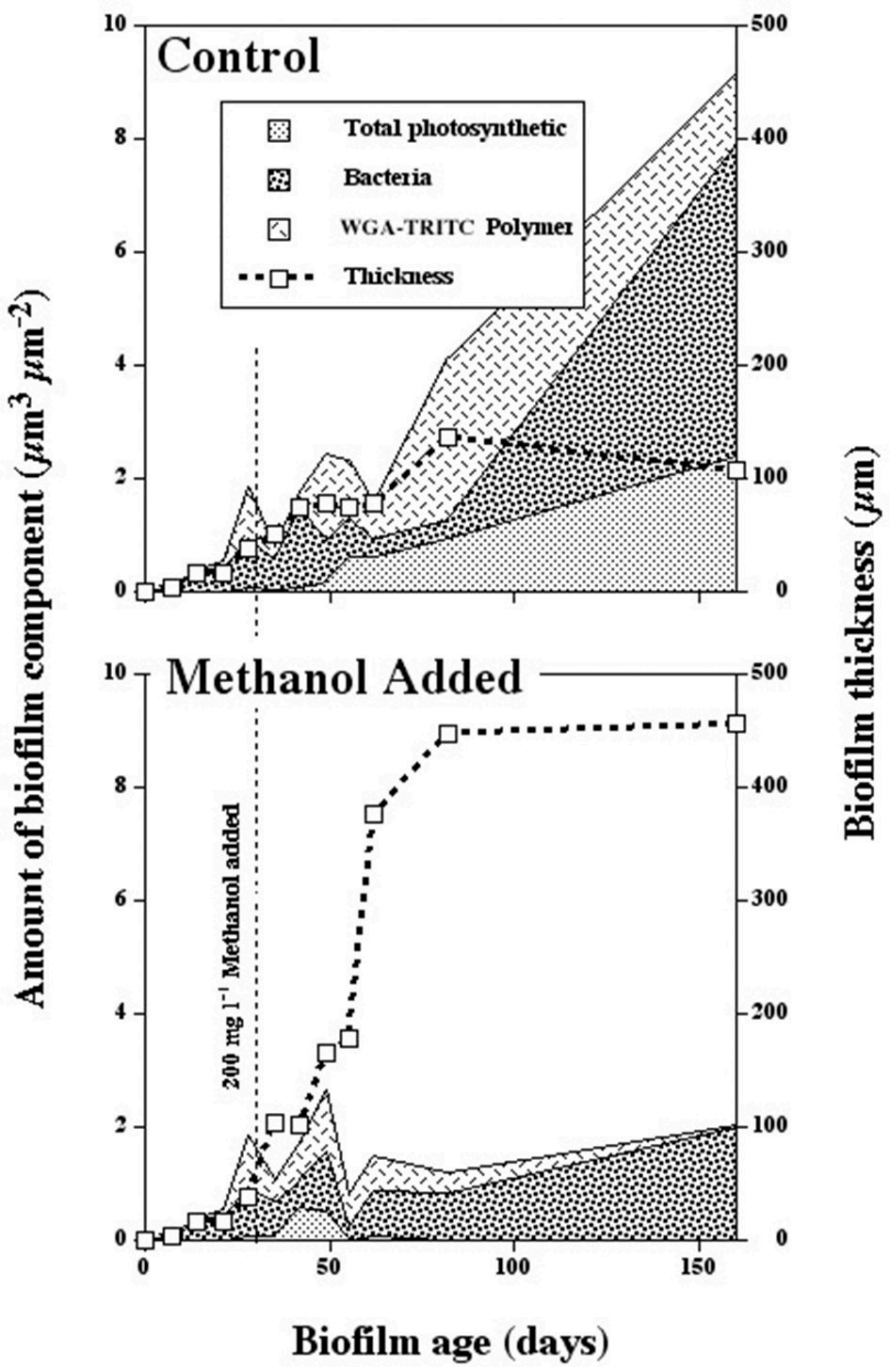
Graphs showing the results of digital image analyses of confocal images indicating the changes in bacteria, total photosynthetic biomass (algae and cyanobacteria), exopolymeric substances and biofilm thickness in river biofilms without (top) and with (bottom) methanol additions.

When river biofilms were exposed to 200 mg l^−1^ methanol after 30 days of development, there was a rapid divergence in developmental pathway characterized by the loss of photosynthetic biomass, changes in exopolymer production and biofilm thickness as well as altered macroscopic appearance of the biofilm. Figures 1A–F, shows a series of CLSM images illustrating the change in biofilm appearance and community composition in the methanol fed vs. reference systems. The loss of algal biomass (red), increase in bacterial abundance (green) and a loss of *Triticum vulgaris* binding sites in the exopolymer (blue) is evident in the 3 channel projections. An examination of the macroscopic appearance of the biofilm (Figures 2A,B) clearly shows the differences between the methanol fed and reference biofilms in terms of physical appearance, thickness and quantity of exopolymeric substances. The methanol biofilms were up to 4,000 μm in thickness, colorless, opaque and without evident inclusions. In contrast, the reference biofilms were less than 200 μm thick, pigmented and with visible algal inclusions. The results of digital image analyses (Figure 3) shows the significant changes in bacteria, photosynthetic biomass, exopolymeric substances and biofilm thickness after the initiation of methanol additions. Reference biofilms continued to develop at the interface reaching a maximum thickness of 100+ μm by 90 days, whereas the methanol treated biofilms rapidly increase in thickness reaching an average thickness of 450 μm by 90 days which was sustained to the end of microscopic analyses. Of particular interest was the change in exopo1ymer; whereas *Triticum vulgaris* lectin detected increasing quantities of exopo1ymer in the reference biofilms it detected virtually no exopo1ymer in methanol fed biofi1ms by 160 days. This is consistent with a change in the composition of the exopo1ymer rather than its loss from methanol biofilms (see below). Similarly, the photosynthetic biomass of the reference biofilm increases over the analytical period whereas it disappears after 60 days in the methanol treatment. Table 2 provides a statistical analysis of the data sets for the reference and methanol biofilms. There was a statistically significant increase in algal biomass immediately after methanol addition followed by a significant decline. Bacterial biomass exhibited both significant increases and decreases in the methanol biofilm vs. the reference system. Similarly, *Triticum vulgaris* binding sites increased initially in the methanol biofilms but ultimately declined as EPS composition changed. In contrast, the overall biofilm thickness was significantly greater in the methanol fed biofilm throughout the analysis period (Table 2). Periodic sloughing events were visually observed during the reactor operation interval of >2 years. Figure 3 documents three of these events in the methanol treatment which occurred at 35, 62, and 90 days and 2 events in the reference at 35 and 62 days. These events occurred when biofilm material detached from the reactor surfaces resulting in a patchy distribution of biofilm on the removable polycarbonate coupons. Reactor evaluation (Lawrence et al., 2000) and additional studies would suggest that these patterns of community response would be highly repeatable (Lawrence et al., 2005; Neu et al., 2005).

**Table 2:** Results of statistical comparison of reference and methanol fed biofilm parameters during the developmental period (160 days).

					**WGA-TRITC**	**WGA-TRITC**		
	Total photosynthetic biomass^**^		**Bacteria**	**Bacteria**	**Polymer**	**Polymer**	**Thickness**	**Thickness**
**Biofilm age**	**(**μ**m**^3^**/**μ**m**^2^**)**	**(**μ**m**^3^**/**μ**m**^2^**)**	**(**μ**m**^3^**/**μ**m**^2^**)**	**(**μ**m**^3^**/**μ**m**^2^**)**	**(**μ**m**^3^**/**μ**m**^2^**)**	**(**μ**m**^3^**/**μ**m**^2^**)**	**(**μ**m)**	**(**μ**m)**
**(days)**	**Reference**	**Methanol**	**Reference**	**Methanol**	**Reference**	**Methanol**	**Reference**	**Methanol**
0	0.00		0.00		0.00		0	
7	0.06		0.07		0.00		5	
14	0.01		0.31		0.10		17	
21	0.02		0.35		0.21		18	
28	0.09		0.87		0.90		39	
30	200 mg l^−1^ methanol treatment starts							
35	0.05	0.10	0.54	0.63	0.23	0.34	52	110^*^
42	0.10	0.60^*^	1.4	0.47^*^	0.30	0.66^*^	75	100^*^
49	0.22	0.54	0.71	1.1	1.5	1.1	78	170^*^
56	0.65	0.04^*^	0.67	0.19	1.0	0.59	75	180^*^
62	0.64	0.09^*^	0.33	0.81^*^	0.61	0.61	78	380^*^
82	0.96	0.01^*^	0.33	0.83^*^	2.8	0.37^*^	140	450^*^
160	2.4	0.00 nd	5.5	2.0 nd	1.3	0.05 nd	110	460 nd

* *Significantly different from corresponding reference value @p ≤ 0.05*.

** *Total photosynthetic biomass includes algae and cyanobacteria*.

*nd, not determined*.

### Community composition

**Figure 4:**
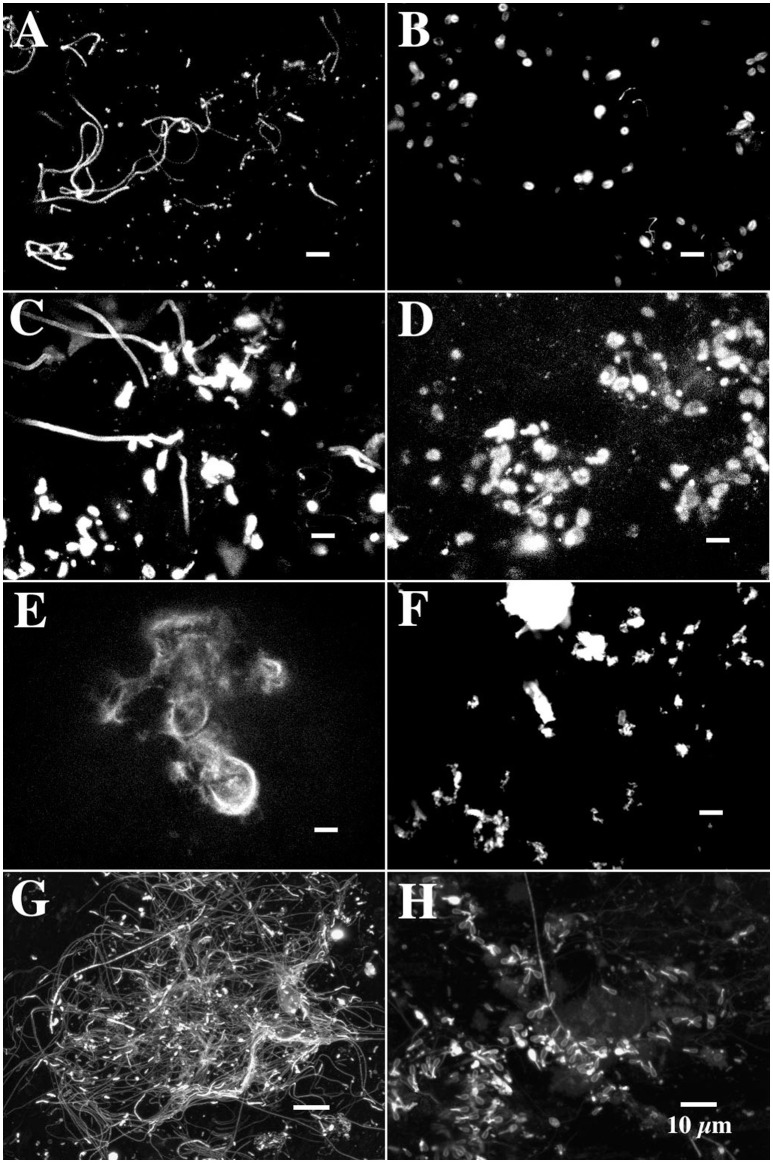
**(A–H)** CLSM micrographs showing the binding of a panel of fluor conjugated lectins within methanol biofilms at 100 days. **(A)** *Arachis hypogaea* conjugated to CY5 binds to the cell surfaces of filamentous, rod and cocci shaped cells in the matrix, **(B)** *Canavalia ensiformis* (TRITC) binds to EPS surrounding a specific cell type in the biofilms, **(C)** *Wisteria floribunda* (FITC) binds to surfaces of large filaments and small colonies, **(D)** binding of *Tetragonolobus purpureas* (CY5) to polymer associated with the globular structures (at arrow) in the biofilm, **(E)** *Ulex europeaus* (FITC) binds to structural polymer similar to that seen in Figure 5C, **(F)** lectin from *Caragana arborescens* displayed binding to small masses of polymer. **(G–H)** CLSM micrographs of dual labeled (SyproOrange and *Helix aspersa-*FITC*)* methanol biofilm showing the diversity of bacterial morphologies found in interstitial regions of the matrix.

Conventional plate count and most probable number analyses confirmed the existence of a physiologically diverse community in the biofilms. These techniques indicated enrichment of populations of nitrifiers, denitrifiers and methylotrophic bacteria (10^2^, 10^4^, 10^3^ cm^−2^ respectively) relative to reference biofilms (Table 3). Fluorescence *in situ* hybridization with a panel of group specific probes indicated that the community was dominated by alpha-proteobacteria, and cytophaga-flavobacterium probe positive bacteria, whereas the beta, gamma proteobacteria and SRB positive cells were minor constituents (Table 4). Application of specific probes for *Paracoccus* indicated the presence of these bacteria in the biofilm; however they were distributed throughout the matrix and rare (< 1 %). Application of fluorescent stains (Day 160) revealed a greater diversity of bacterial cell types within the interstitial polymer. For example, as shown in Figures 4 A–H, *Arachis hypogeae-CY5* bound to cell surfaces of filamentous, rod and coccoid shaped cells, *Canavalia ensiformis-FITC* bound to other cell types, while *Wisteria floribunda-* FITC bound to large filaments and small colonies in the interstitial matrix. The hydrophobic dye Nile Red bound to cell clusters within the interstitial matrix (Figure 4). The protein sensitive stain, SyproOrange revealed a diversity of bacterial morphotypes including club-shaped cells within the interstitial polymer (Figures 4G,H). However, the dominant structural and microbiological feature of the methanol biofilm community was the “globular” structures distributed within the interstitial polymer matrix.

**Table 3:** Determination of the relative abundance of selected microbial populations in reference and methanol fed biofilms, as detected by plate count and MPN methods.

	MeOH^*^	Reference^*^
Total heterotrophs (10% TSA)	3.0 × 10^6^	1.7 × 10^5^
Actinomycetes (Actinomycete Isolation Agar)	3.9 × 10^5^	1.5 × 10^4^
Pseudomonads (Pseudomonas Isolation Agar)	9.6 × 10^1^	5.2 × 10^1^
Fungi (Rose Bengal Agar)	2.7 × 10^2^	7.8 × 10^2^
Methylotrophs (Methylotroph Methanol medium)	1.9 × 10^3^	1.7 × 10^2^
NH_4_ Oxidizers	ND	1.4 × 10^2^
NO_2_ Oxidizers	1.4 × 10^2^	9.7 × 10^1^
Denitrifiers	4.0 × 10^4^	3.1 × 10^3^
Sulfur Oxidizers (ATCC 125 Medium)	2.1 × 10^2^	6.3 × 10^1^
Iron Oxidizers (K9 Mineral Salts Medium)	1.1 × 10^1^	ND
Sulfate Reducing Bacteria (Peptone Iron Agar)	3.9 × 10^3^	3.5 × 10^3^

* *Colony forming units or MPN cm^-2^, ND, none detected*.

**Table 4:** Results of 16S r-RNA probe analysis of the methanol biofilm.

**Probe**	**Percent area**	**Percent of total counts**
Total (Sytox)	1.08 ± 0.58	
Eubacteria (EUB 338)	0.76 ± 0.46	70 ± 43
Alpha 1b	0.34 ± 0.15	32 ± 14
Beta 42a	0.18 ± 0.14	16 ± 13
Gamma 42a	0.15 ± 0.10	14 ± 9
CF 319a	0.36 ± 0.33	34 ± 31
SRB 385Db	0.15 ± 0.11	14 ± 10

### Reactor operation

The methanol fed reactor, with a flow through rate of 1.0 1 d^−1^, and low nitrate levels, NO_3_-N levels were reduced 95% (N from 0.187 mg 1^−1^ to 0.01 mg 1^−1^) while total phosphorous was reduced by 70% (TP from 0.02 mg 1^−1^ to 0.005 mg l^−1^) and carbon by 24% (from 56.1 mg 1^−1^ to 42.8 mg 1^−1^). In contrast, the reference reactor resulted in reductions of N 95% *(N* from 0.19 mg 1^−1^to 0.01 mg 1^−1^) while total phosphorous was reduced by 90% (TP from 0.02 mg 1^−1^ to 0.002 mg 1^−1^) and carbon by 0% (from 3.1 mg 1^−1^ to 3.2 mg 1^−1^). Thus both reactors functioned to reduce N and P levels in the exit waters to near detection levels.

### Internal biofilm structure

**Figure 5:**
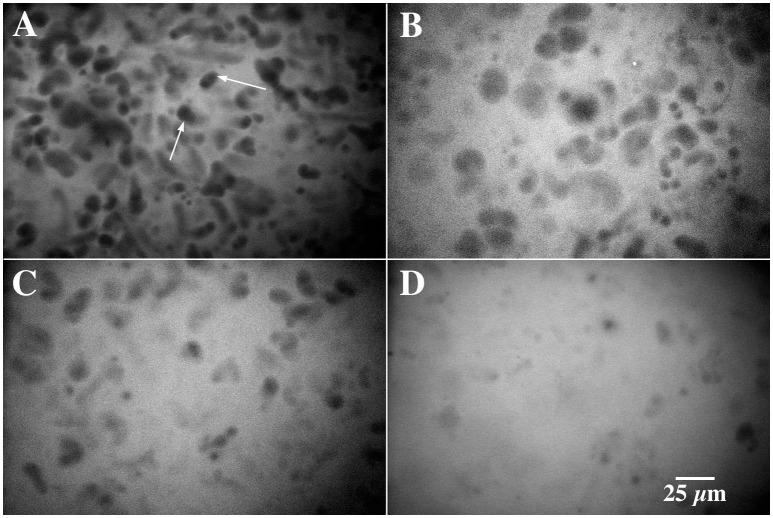
**(A–D)** A series of CLSM micrographs showing the penetration of **(A)** 2,000K molecular weight (m.w.), **(B)** 400K m.w., **(C)** 40K m.w., and **(D)** 4K m.w. FITC conjugated dextrans into a methanol biofilm. Arrows indicate the location of the negatively stained globular structures (dark regions) surrounded by more permeable matrix polymer (see also Supplemental Movie 1).

**Figure 6:**
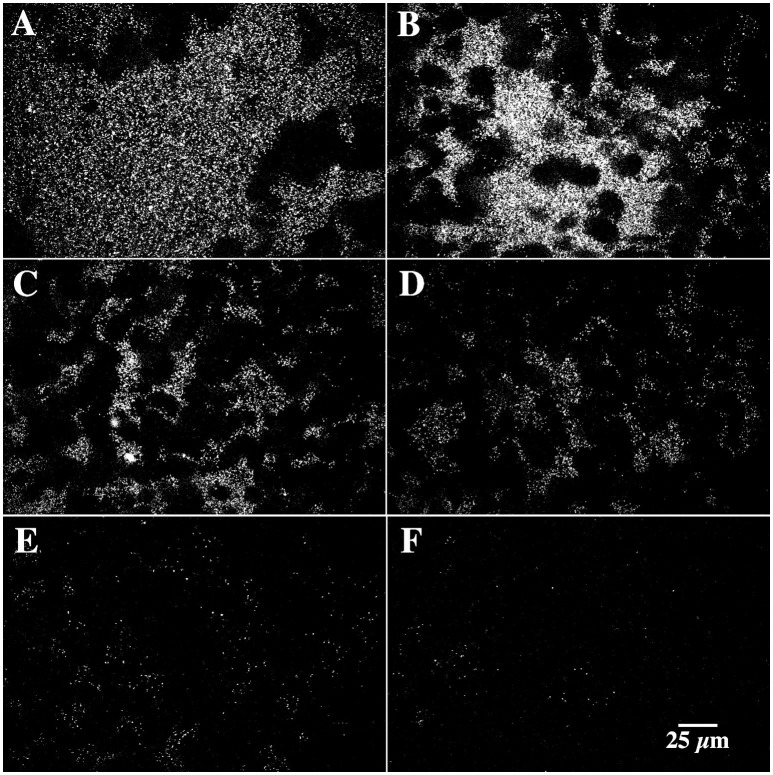
**(A–F)** A series of laser micrographs showing the penetration of 0.2 μm fluorescent latex beads into 400 μm of methanol biofilm material. Note that the beads, penetrate 400 μm of biofilm thickness, and that there are regions where the beads are excluded **(B,C)** which correspond the outline of the globular microcolonies.

**Figure 7:**
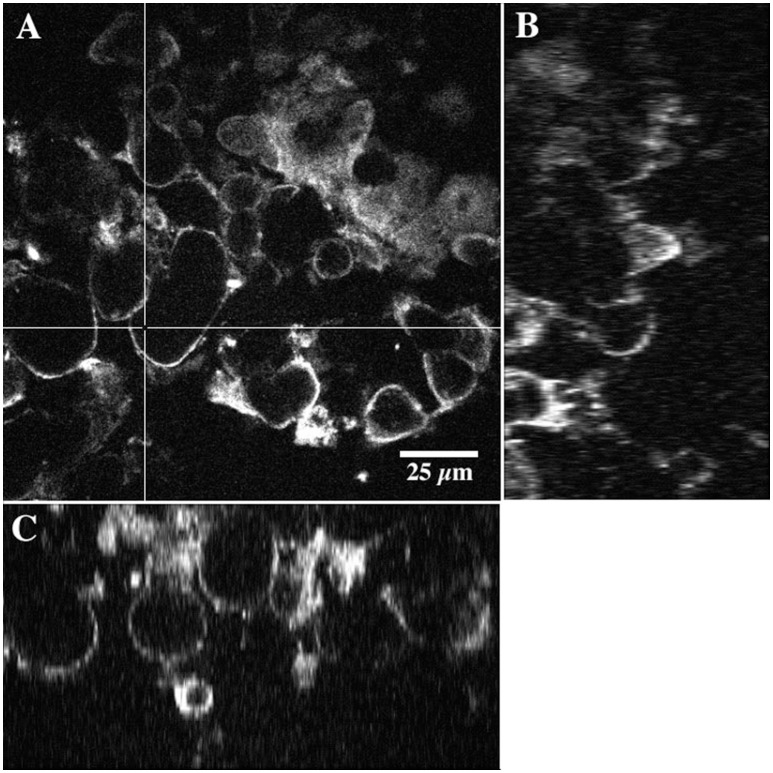
A series of 2-photon micrographs showing details of the nature of the boundaries of the globular structures in the methanol biofilm community. 160 day old specimen was labeled with *Ulex europeaus-FITC*. **(A)**, xy image showing locations (crossed lines) of the two xz images shown in image **(B)** and **(C)** (see also Supplemental Movie 2).

**Figure 8:**
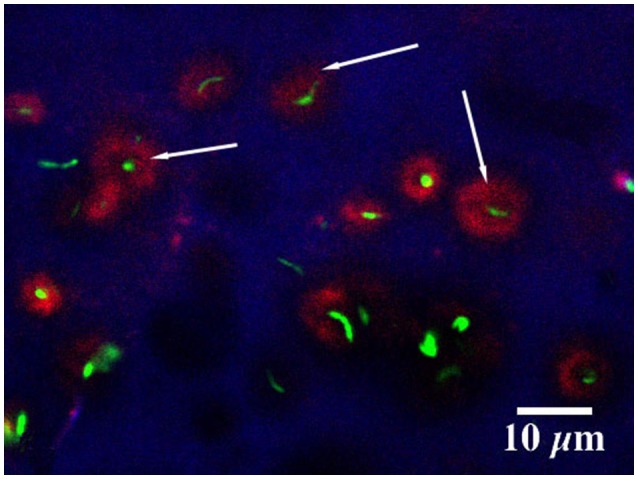
CLSM three channel image showing the staining of cells with the nucleic acid stain Syto 9, (green) the 70K m.w. polyanionic dextran (blue) and *Tetragonolobus purpureas* lectin (CY5) (red). The images show the presence of bacterial cells in the globular structures (at arrow) and a unique polymer (red) surrounding the cells (green), with an interstitial polymer of differing charge and chemistry (blue).

**Figure 9:**
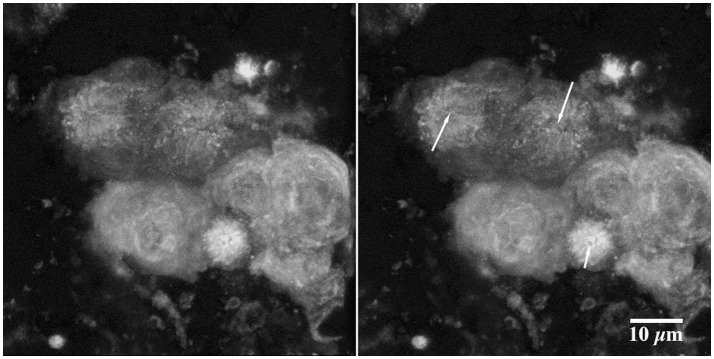
2P-LSM stereo projection of optical sections through globular structures stained with *Ulex europeaus-FITC*, showing details of the internal structure. The cellular space is indicated by the small arrow while a larger arrow points to the bundle structure of the surrounding polymer. Images are best viewed with a stereo viewer. (see also movie Supplemental Movie 3).

In the present study the methanol biofilm structure was characteristically an extensive polymer matrix with included distinct globular structures or microcolonies. These structures appear negatively stained in CLSM images taken after the addition of 2000K, 400K, 40K, and 4K molecular weight dextrans conjugated to fluorescein (see arrows Figure 5). See also Supplemental Movie 1 providing a fly-through animation of a confocal image stack using 2,000K dextrans. Penetration appeared to increase with decreasing molecular weight or radius of gyration with the greatest penetration of the structures occurring with 4K dextrans (Figure 5). The application of 0.2 μm fluorescent beads to the methanol biofilms also shows the presence of these globular structures in the biofilm mass (Figures 6 A–D). These appear similar to the images obtained with dextrans (Figure 5). Further they indicate that the interstitial polymer is porous allowing migration of 0.2 μm beads to a maximum depth of 500 μm in a total depth of 1,000 μm. Detailed examination of these globular structures using 2-Photon laser microscopy and the fucose sensitive lectin *Ulex europeaus-FITC* indicated that they had an external polymer layer containing fucose residues. This structure can be seen in both xy and xz or sagittal section images of the structures shown in Figures 7A–C. See also Supplemental Movie 2 providing a 2P-confocal xy and xz fly-through animation of an image stack stained with the lectin *Ulex europeaus-FITC*. Combined application of Syto9, 10K polyanionic dextran and *Tetragonolobus purpureas-CY5* lectin provided the most comprehensive imaging of the interstitial polymer, globular structures and enclosed individual cells (Figure 8). In this 3 channel image stack, the globular structures can be seen to contain one or two bacterial cells (green) surrounded by the fucose-rich *(Tetragonolobus purpureas-CY5* lectin binding) polymer (red) which is in turn enclosed by the interstitial polymer which binds the polyanionic dextran (blue). Figure 9 shows a detailed examination of several globular structures stained only with *Ulex europeaus-FITC* lectin, imaged at 2 μm intervals and projected as a stereo pair. The internal structure appears to contain bundles of polymer fibrils enclosing a central space (at arrow) which would be occupied by an individual bacterial cell as shown in Figure 8. See also Supplemental Movie 3 providing a high resolution confocal xy fly-through animation of an image stack stained with the lectin *Ulex europeaus-FITC*. These observations confirmed that there were bacterial cells within the globular structures which had a unique exopolymer which was further surrounded by an interstitial polymer of differing charge and chemistry.

### Analyses of EPS chemistry

**Figure 10:**
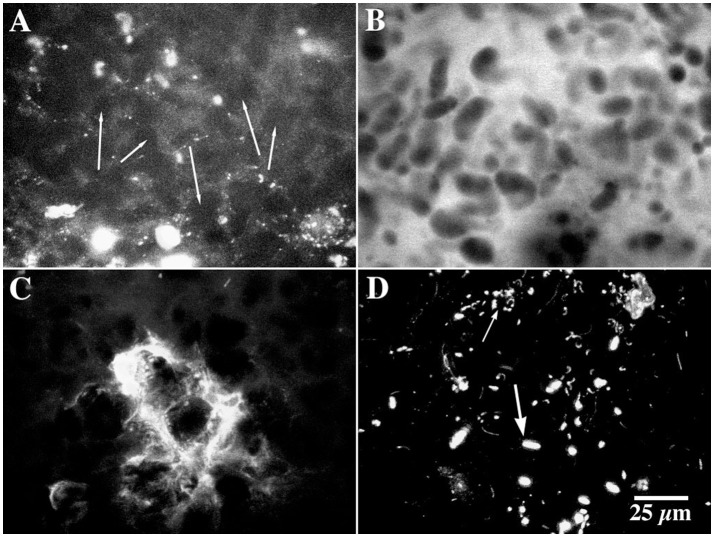
**(A–D)** CLSM micrographs showing the effect of charge on the distribution of fluor-conjugated dextrans in the methanol biofilm. **(A)** neutral charge 10K m.w., rhodamine B conjugated dextran which binds to diffuse polymer regions between the globular structures (at arrows), **(B)** an anionic FITC conjugated 10K m.w., dextran, which binds more extensively to the diffuse polymer regions (at arrow), **(C)** binding of a polyanionic 10K m.w. dextran to a fibrous polymer region (at arrow) surrounding the globular structures, **(D)** binding of the hydrophobic dye Nile red to cell surfaces and cell clusters (at arrow) in the interstitial region surrounding the globular structures (at large arrow). (see also Supplemental Movie 4).

Chemical analyses of the total polymer indicated that the dominant neutral sugars were glucose, galactose, mannose, rhamnose, with fucose and ribose as minor constituents. Amino sugars were not detected (Table 5). Additional *in situ* probe analyses indicated that the globular structures bound, FITC and CY5 conjugated lectins, from *Canavalia ensiformis, Tetragonolobus purpureas, Ulex europeaus*, and *Arachis hypogaea*. In contrast the interstitial polymer bound *Ulex europaeus* but not *Tetragonolobus purpureas*, and in addition bound the lectins from *Lens culinaris, Triticum vulgaris, Vicia faba*, and *Wisteria floribunda*. The FITC-lectin of *Phaseolus vulgaris* bound both the globular structures and more generally within the matrix (Table 1). The nature of the charge distribution and hydrophobicity within the biofilm were also investigated using CLSM and a panel of fluorescent probes. In addition to the extensive binding of the 10K mw polyanionic dextran, a neutral 10K mw dextran bound to regions of the interstitial polymer between the globular structures (Figures 10 A,B). This dextran also bound to a fibrous polymer region observed at the surface of the globular structures (Figure 10C). Fibrous regions are well illustrated by the appearance of staining in Figure 10C where the polyanionic dextran has bound to a more fibrous region surrounding the globular structures. Various aspects of the globular structures are shown in Figures 5–7, 9, 10. See also Supplemental Movie 4 which shows aspects of Figures 4, 10 in a 3D stack animation.

**Table 5:** Relative abundance (percentage) of assorted neutral sugars in the methanol fed biofilm and reference river biofilm determined by gas chromatography and mass spectroscopy.

**Treatment/Neutral sugar**	**Rhamnose**	**Fucose**	**Ribose**	**Arabinose**	**Fructose/Mannose**
Reference	4	6	10	10	2
Methanol	7	2	2	1	6
**Treatment/Neutral sugar**	**Glucose**	**Sorbose**	**Glucosamine**	**Galactosamine**	**Total**
Reference	68	0	0	0	100
Methanol	82	0	0	0	100

### Gradient detection

**Figure 11:**
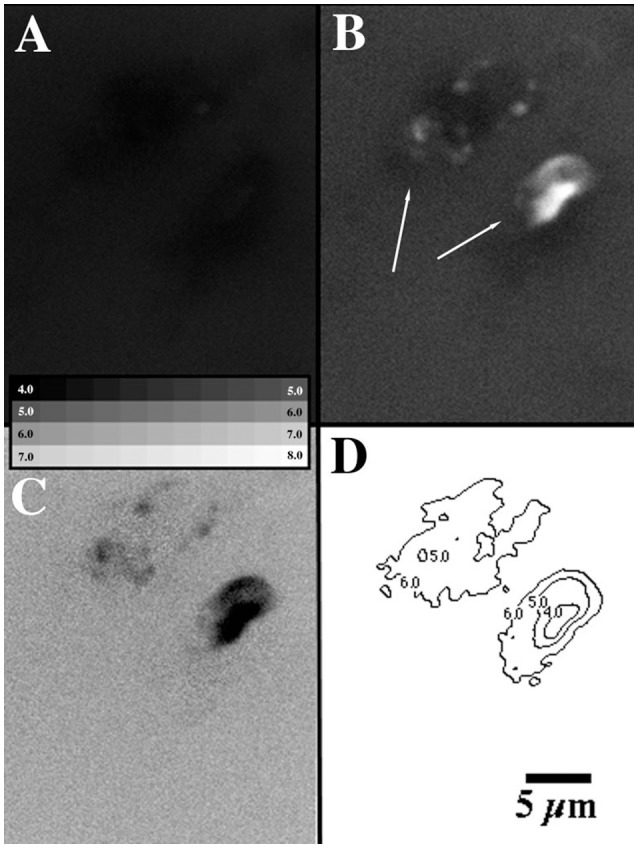
**(A–D)** CLSM micrographs illustrating the existence of pH gradients within the methanol biofilms. **(A)** rhodamine signal, **(B)** fluorescein signal, **(C)** ratio image, **(D)** contour map of pH.

Application of a Pt wire micro electrode did not detect changes in Eh within the biofilm over a depth of 3000 μm at multiple (*n* = 10) locations. Eh values remained relatively constant in the range of +500 + 10 mV throughout the biofilm regardless of xy or xz location. Figure 11 illustrates the detection of a pH gradient between the inside and outside of the globular structures in the interstitial matrix. The pH sensitive dye fluorescein and pH insensitive dye rhodamine are simultaneously imaged at the same location in the biofilm. The result of the ratio of these images (Figure 11C) with two globular structures (at arrows) indicates a reduction in pH within the globular structures. Application of a standard curve created from fixed equilibrated biofilm material allows an estimation of the pH in the interstitial region at pH 7.0 vs. as low as 4 to 5 within the globular structures (Figure 11D).

## Discussion

### Biofilm development

The role of biofilm architecture in the function of aerobic degradative communities has been established by a number of studies including Wolfaardt et al. (1994, 1998), Massol-Deyá et al. (1995), Macedo et al. (2005, 2007) and Wouters et al. (2010). It has been suggested that these structural traits are important in biofilm function and optimization of degradative processes in biofilms. Here we described the development of a methanol fed community when river water is the source of inoculum and of general nutrients.

Evaluation of methanol biofilms by Neef et al. (1996) indicated that those growing on sand grain supports were characteristically 10–50 μm thick with a median of 20 ± 12 μm. They added 30 to 135 mg 1^−1^ methanol whereas in our study the methanol loading rate was set at 200 mg 1^−1^. Neef et al. (1996) reported the presence of a variety of denitrifiers including *Paracoccus* spp., while other authors have reported members of the genera *Alcaligenes, Hyphomicrobium and Pseudomonas*, and enrichment of methylotrophic bacteria (Knowles, 1982). The enrichment of nitrifiers, denitrifiers and methylotrophic bacteria (Table 3) was consistent with that reported for other methanol fed biofilm systems (Lemmer et al., 1997b). The relatively low nitrate levels in our river water fed system likely would have mitigated against the proliferation of denitrifiers. Methylotrophs are generally favored by the provision of C 1-subtrates such as methanol. In our study, the dominance of alpha-proteobacteria was consistent with the substantial number of methanol utilizing genera within this subgroup (e.g., *Paracoccus* spp. *Hyphomicrobium* spp.). However, river biofilms without methanol additions were also dominated by alpha-proteobacteria and cytophaga-flavobacterium positive cells (Manz et al., 1999). Whereas, Neef et al. (1996) reported that their biofilms were dominated by Bet42a positive cells which were 60 to 70% of total EUB338 positive cells, and the Alf1b group probe only hybridized with 10–15% of EUB338 positive cells, with 40–50% of these being *Paracoccus* spp. In contrast, while the PAR 651, 1244, and 1457 probes revealed the presence of *Paracoccus* cells and cell clusters within our methanol fed biofilms they were a rare member of the community constituting < 1% of total cells. In comparison, Lemmer et al. (1997b) reported that the genus *Paracoccus* alone constituted 3–5% of all cells. Thus the methanol fed biofilm community in the present study differed markedly from those previously described. This may be attributed to the lack of high nitrate loading in our case (0.19 mg NO_3_-N l^−1^ vs. 10 to 45 mg NO_3_-N l^−1^ and phosphate loading of 0.02 mg l^−1^ vs. 0.1–0.7 mg l^−1^ in the study of Neef et al. (1996). Despite the lack of N loading, methanol additions did contribute to enrichments of bacterial populations in the reactors including those of nitrifiers and denitrifiers (Table 3).

Also of interest was the observed loss of photosynthetic biomass in the methanol fed reactors after their initial establishment in the biofilm community. Verschueren (1983) reviewed the biological effect of methanol and reported the following effects, reduction of sludge digestion at a concentration of 800 mg l^−1^ and NH_3_ oxidation (50% inhibition) in *Nitrosomonas* at a concentration of 160 mg l^−1^. In general, methanol is not considered highly toxic, reports for a wide variety of organisms indicated a cessation of cell division only occurs at hundreds or thousands of mg l^−1^ methanol. For example, cell division ceased for *Pseudomonas putida* 6,600 mg l^−1^, *Microcystis aeruginosa* 530 mg l^−1^, *Scenedesmus quadricauda* 8,000 mg l^−1^, *Entosiphon sulcatum* >10,000 mg l^−1^ (Verschueren, 1983). Our observation is therefore surprising and may indicate the operation of community level interactions that influence the survival of phototrophs in the presence of methanol.

### Reactor operation

The present system was not subjected to high or typical waste stream loading of nitrate or phosphorus where the goal may be to reduce inorganic N levels to 18 mg l^−1^ (German legislative standard). Lemmer et al. (1997a) used a methanol fed reactor with a quartz sand carrier and 20 μm thick biofilms, to reduce influent nitrate concentrations from 25 mg 1^−1^ to 0–2 mg l^−1^. In our system, with a flow through rate of 1.0 l d^−1^, a 450+ μm thick biofilm system and low nitrate levels, NO_3_-N levels were reduced 95% while total phosphorous was reduced by 70% and carbon by 24%. The reference river water reactor had a similar effect on N and P but no detectable effect on the level of carbon measured in the waters. In the described set up both reactors are likely N and P limited.

### Internal biofilm structure and chemistry

The results of the mass spectrometry analyses (Table 5) confirmed the presence of fucose residues and an abundance of glucose in the exopolymer of the methanol biofilm community. Lectin binding analyses also indicated the presence of these residues and suggest that the fucose residues are localized in the globular structures, with glucose being abundant in the interstitial matrix polymer. A comparison of the mass spectrometry analyses for reference and methanol fed biofilms indicated that although the composition was similar in both cases, substantial increases in rhamnose and glucose sugars occurred in the methanol fed system. Based on the lectin binding analyses and observed increase in mass, these sugars were most likely abundant within the interstitial polymer. This is the first instance to the author's knowledge of successful correlation of the results of mass spectrometric analyses with the results of *in situ* lectin analyses and localization of glycoconjugates in the biofilm.

Lectins have been used as a probe of microbial extracellular polymers in a number of studies of environmental biofilm systems (Michael and Smith, 1995; Neu and Lawrence, 1997; Wolfaardt et al., 1998; Neu, 2000; Neu et al., 2001; Lawrence et al., 2016; Pronk et al., 2017). These studies have similarly shown that a diversity of lectin binding sites could be detected within microbial biofilms using CLSM, and that they may be associated with specific community members. Other studies have also indicated that the carbon source may dramatically influence composition and nature of the exopolymer matrix of microbial communities (Bonet et al., 1993) and may alter the community structure of river biofilms as well as the quantities of exopolymer produced (Mohamed et al., 1998; Lawrence et al., 2005; Neu et al., 2005). For example, carbon source dramatically influenced the glycoconjugate makeup of a degradative biofilm community as detected by lectin analyses (Wolfaardt et al., 1998). In this instance the shifts were attributed to changes in the relative abundance of community members. Shifts have also been observed in response to various nutrients, chemical agents and stressors (Lawrence et al., 2005; Neu et al., 2005). Thus the shifts in glycoconjugate make up observed in the methanol biofilm are in keeping with trends observed in a variety of other biofilm systems.

Various aspects of the globular structures are presented in Figures 5–7, 9, 10. The consensus description suggests ovoid structures with a multilayered exopolymer rich in fucose residues, the outer most layer appears fibrous and embedded in the surrounding interstitial polymer. The boundary of the globular structures is diffuse but not penetrated by fluorescent probes with a hydrated radius greater than approximately 10 nm whereas the interstitial matrix polymer allows free migration of 200 nm beads. In keeping with these observations, Lawrence et al. (2016) reported that a beta-proteobacterial microcolony intercellular EPS were permeable to beads that were ≤ 40 nm in diameter and dextrans with a hydrated radii of 1 to 10 nm. These structures are not similar to those described by other authors for systems with abundant *Paracoccus* populations. Neef et al. (1996) described dense aggregates of coccoid cells which occurred in clusters of several 100 cells. In addition, *in situ* probing did not result in detection of *Paracoccus* or members of other specific groups within the globular structures.

### Microenvironmental conditions

The presence of channels and pores as a significant part of biofilm architecture was first noted by Lawrence et al. (1991). These structural features have now been reported from a number of aerobic biofilm systems and are featured in current “models” of biofilm structure and function. Massol-Deyá et al. (1995) reported cell free channels which interconnected the surface film in their biofilm reactors within the deep inner layers. In toluene fed biofilms of up to 300 μm thickness they reported that a large number of vertical and horizontal channels extended into at least one half the depth of the film. Kloep et al. (2000) found a very heterogeneous biofilm structure in a nitrifying biofilm system where the biofilm varied between 12 and 166 μm in thickness and was characterized by channel structures oriented in the longitudinal direction of the carrier material. We observed that the methanol biofilm matrix was extensively penetrated by a fine fissure network as revealed by the location of fluorescent (200 nm) latex beads detected by confocal laser microscopy (Figure 6). In keeping with the report of Massol-Deyá et al. (1995) we found that these beads penetrated to at least one half the biofilm depth. It was also evident that these beads did not penetrate into the globular structures in the biofilm. The mobility of various size-fractionated fluor conjugated dextrans indicated that the basal region of the biofilm was only penetrated by the lowest molecular weight probes with a hydrated radius of 2 nm. Similar patterns of size fractionated exclusion of fluorescent probes by biofilm materials have been described previously (Lawrence et al., 1994; De Beer et al., 1997). Lawrence et al. (2016) reported similar observations at the microcolony scale. This suggested that hindered diffusion occurred within the biofilm (particularly in the region of the globular structures) but that channels connected the biofilm mass to the bulk phase of the reactor. These channels may act to increase the biofilm surface area per unit volume, which would facilitate transport into and out of the biofilm matrix, overcoming diffusion limitation of biofilm metabolism and maintaining an aerobic environment (as indicated by Eh measurements) despite biofilm thickness. In addition the pH outside the globular structures was found to be in the range of 7 throughout the biofilm. In general, it has been suggested that thick biofilms which would promote anaerobic conditions with concomitant production of acids and sulfides and metabolic intermediates should be strictly avoided (Lemmer et al., 1997a). Further the associated reduction in pH is also considered deleterious to nutrient consumption through nitrification and denitrification processes. However, as shown in the current study, it is evident that development of specific biofilm architecture may overcome these potential limitations allowing maintenance of optimal conditions in the biofilms. Therefore we investigated micro environmental conditions in the biofilm system. Other authors have utilized interstitial fluids to investigate reactor conditions rather than examining biofilm micro environmental conditions *in situ*. They reported that oxygen ranged from 4 mg l^−1^ to undetectable with increasing depth in the reactor, that redox ranged from 300+ to 100+ mV in the same interval and that pH was generally in the range 6.5–6.9 (Lemmer et al., 1997a). In some cases studies have applied microsensors to demonstrate the presence of steep oxygen gradients in relatively thin aerobic biofilms (DeBeer et al., 1994). In a study of nitrifying biofilm reactors (Schramm et al., 1999a, b) reported that all oxygen was consumed in the upper 50–100 μm of the biofilm and that aerobic nitrification processes were limited to this zone. In the present study we tested for the presence of gradients within the biofilm using Pt wire microelectrodes. However, we did not detect changes in Eh within the biofilm over a depth of 3,000 μm. Eh values remained relatively constant in the range of +500 mV throughout the biofilm regardless of xy or xz location. Given observations on apparently thinner biofilms with less available carbon (Schramm et al., 1999b) these results were surprising.

The appearance of negative staining (Caldwell et al., 1992) may also be consistent with the presence of low pH (< pH 5) within the globular structures. Fluorescein is known to be quenched in the presence of hydrogen ions, with no fluorescence below pH 5 and increasing fluorescence to pH 9. Thus the observations in Figure 5 are consistent with reduced pH micro sites within the methanol biofilms. To investigate these gradients further a dual labeled rhodamine fluorescein dextran was added to the biofilm and ratiometric imaging carried out. Rhodamine is pH insensitive in terms of fluorescence whereas fluorescein is pH sensitive (see above). The results of this analysis (Figure 11) indicated that the globular structures had a reduced interior pH. This indicated the presence of clearly defined micro environments within the globular structures relative to the interstitial matrix which appeared to have a generally neutral pH. Vroom et al. (1999) also reported the detection of pH gradients within relatively thick dental biofilms using laser microscopy. They detected pH gradients in both the lateral and axial directions, in steady-state biofilms. Further addition of 14 mM sucrose for 1 h, resulted in distinct pH gradients where microcolonies with pH values of below pH 3.0 were visible next to areas with higher pH (>5.0). Similarly, Lawrence et al. (2016) described steep pH gradients within microcolonies. In this case the pH gradient went from the cell (pH 7) to the intracellular region with pH 4+ increasing to pH 7+ at the colony exterior boundary. In the current study gradients from pH 4 to 7 occurred over a similar spatial scale of < 5 μm. Thus the architectural arrangement of the biofilm and the nature of its permeability and porosity described above allowed for the development of microscale variation associated with the globular structures but apparently not within the interstitial matrix of the biofilm. The literature indicates that maximum thicknesses for methanol biofilms in reactors may be 20 μm for optimal operation (Neef et al., 1996). Our observations indicate that this maximum thickness may be greater and is dependent upon the specific biofilm architecture. The EPS is clearly the critical element in biofilm architecture and it structures both the physical and chemical environment at the micro- or microcolony scale as well as in an integrated way within the overall biofilm. Although it is possible that physical mechanisms play a role in the formation of these architectural features it is more likely that as described by Weissbrodt et al. (2013) for granulation, interactions between dominant microbial populations are essential for optimization of the microenvironment and metabolic processes. These observations reinforce the critical role of microscale observations on living biofilms play in our understanding of these complex systems.

## Summary

Integration of the information obtained by microscale analyses indicates that these methanol fed biofilms exhibited an architecture dominated by a chemically and structurally complex globular microcolony which maintained a unique microenvironment. In contrast, the interstitial matrix was extensively penetrated by a porous network. This biofilm architecture resulted in the unhindered migration of cellular waste and nutrients facilitating the development of extensive thick biofilm communities. This suggests that depending upon the biofilm architecture reactor operations may be optimal at biofilm thicknesses exceeding those reported for some operational reactors, traditionally in the range of 20 μm. All observations are in keeping with self-organization of the biofilm community composition and architecture to optimize growth conditions.

## Author contributions

Experiments, as well as CLSM imaging, microelectrode studies, fluorescent *in situ* hybridization, most probable number (MPN), and plate count estimation of specific microbial populations, were carried out in the laboratory of JL. TN contributed to experimental design and analyses—1P-CLSM analyses, image analyses, and processing. MW contributed to the mass spectrometry of exopolymeric substances. JL, TN, and MW contributed equally to the planning and execution of the experiments and to the preparation of figures, text, etc. for the submitted manuscript.

### Conflict of interest statement

MW is currently employed by Waters Corporation. The remaining authors declare that the research was conducted in the absence of any commercial or financial relationships that could be construed as a potential conflict of interest. The reviewer TM declared a shared affiliation, with no collaboration, with one of the authors, TN, to the handling editor at time of review.
